# Strong in combination: Polyphasic approach enhances arguments for cold‐assigned cyanobacterial endemism

**DOI:** 10.1002/mbo3.729

**Published:** 2018-09-21

**Authors:** Patrick Jung, Laura Briegel‐Williams, Michael Schermer, Burkhard Büdel

**Affiliations:** ^1^ Plant Ecology and Systematics Biology Institute University of Kaiserslautern Kaiserslautern Germany

**Keywords:** Antarctica, Arctic, biogeography, biological soil crusts, cyanobacteria, denaturing gradient gel electrophoresis, endemism, polyphasic approach

## Abstract

Cyanobacteria of biological soil crusts (BSCs) represent an important part of circumpolar and Alpine ecosystems, serve as indicators for ecological condition and climate change, and function as ecosystem engineers by soil stabilization or carbon and nitrogen input. The characterization of cyanobacteria from both polar regions remains extremely important to understand geographic distribution patterns and community compositions. This study is the first of its kind revealing the efficiency of combining denaturing gradient gel electrophoresis (DGGE), light microscopy and culture‐based 16S rRNA gene sequencing, applied to polar and Alpine cyanobacteria dominated BSCs. This study aimed to show the living proportion of cyanobacteria as an extension to previously published meta‐transcriptome data of the same study sites. Molecular fingerprints showed a distinct clustering of cyanobacterial communities with a close relationship between Arctic and Alpine populations, which differed from those found in Antarctica. Species richness and diversity supported these results, which were also confirmed by microscopic investigations of living cyanobacteria from the BSCs. Isolate‐based sequencing corroborated these trends as cold biome clades were assigned, which included a potentially new Arctic clade of *Oculatella*. Thus, our results contribute to the debate regarding biogeography of cyanobacteria of cold biomes.

## 1 INTRODUCTION

Soil habitats occur in rather scattered patterns in polar and Alpine regions of both hemispheres where they share hostile abiotic conditions which include freeze‐thaw cycles, wide irradiance fluctuations, and low nutrient availabilities, resulting in generally depauperate environments and short growing seasons (Bayard, Stähli, Parriaux, & Flühler, 2005; Cary, McDonald, Barrett, & Cowan, 2010; Convey et al., 2014; Cowan, Makhalanyane, Dennis, & Hopkins, 2014; Forman & Miller, 1984; Pointing et al., 2015). Nevertheless, photoautotrophic microbial communities are capable of rapid colonization of even the most extreme terrestrial habitats and function as ecosystem engineers (Belnap & Lange, 2001). Conglomerations of soil particles, cyanobacteria, algae, microfungi, lichens, and bryophytes creating a skin known as biological soil crusts (BSCs) are among these communities and can be found worldwide where higher vegetation is sparse or absent (Belnap, Büdel, & Lange, 2001).

Cyanobacterial communities within BSCs are largely responsible for important ecosystem services such as erosion prevention (Belnap & Gillette, 1998; Bowker, Miller, Belnap, Sisk, & Johnson, 2008), soil formation (Rillig & Mummey, 2006), soil moisture (Belnap, 2006) and carbon‐ and nitrogen cycling (Kowalchuk & Stephen, 2001; Shively, English, Baker, & Cannon, 2001; Tiedje, 1994). As they provide initial structural integrity and possess extremophile characteristics in terms of temperature, freezing and thawing cycle, photoprotection, light acquisition or photosynthesis (Nadeau, Milbrandt, & Castenholz, 2001), they physically modify, maintain or create habitats for other organisms. Thus, cyanobacterial communities are essential components in BSC successional processes and allow further development, usually by the establishment of bryophytes and lichens (Belnap & Lange, 2001). Biological soil crusts of circumpolar habitats have recently been shown to be vulnerable to the potential impact of human induced environmental change as their activity and structure is strongly affected by increasing temperatures or alterations caused by alien plants (Bálint et al., 2011; Escolar, Martínez, Bowker, & Maestre, 2012; Maestre et al., 2013; Pushkareva, Johansen, & Elster, 2016). With each year setting a new low point in global glacier coverage (Zemp et al., 2015), it is imperative that we capture the diversity of cyanobacteria as major ecological components to explain their response to the anthropogenic climate change.

Currently, terrestrial aspects of cold ecosystems with high BSC coverage found at Hochtor (European Alps), Geopol, Ny‐Ålesund (both Arctic, Svalbard), and Livingston Island (Antarctica) have been addressed (Jung, Briegel‐Williams, Simon, Thyssen, & Büdel, 2018; Rippin et al., 2018; Williams, Borchhardt, et al., 2017), but the cyanobacterial community composition received little attention. In Svalbard high bacterial abundance was found in vegetated soils, this was strongly related to the availability of organic matter, inorganic nutrients, and moisture supply (Kaštovská, Elster, Stibal, & Šantrůčková, 2005; Pessi, Lara, et al. 2018; Pessi, Pushkareva, et al. 2018). In contrast, in alpine habitats, the community composition appears to shift markedly along chrono‐sequences, indicating that each soil environment selects for its phototrophic community (Frey, Bühler, Schmutz, Zumsteg, & Furrer, 2013). Antarctic endemism has been interpreted as consequences of the long‐term isolation of the continent from other landmasses, dispersal limitation between isolated ice‐free regions, and the survival of well‐adapted organisms (Fraser, Terauds, Smellie, Convey, & Chown, 2014; Vyverman et al., 2010).

On a local scale, evidence suggests an annual cell circulation among soil, ice, and atmosphere (Broady, 1996; Davey & Clarke, 1991), with wind and water being the transport agents of cyanobacteria entombed in ice and or frozen sediment (Gilichinskii, Wagener, & Vishnivetskaya, 1995; Ponder, Vishnivetskaya, McGrath, & Tiedje, 2004). The frozen habitats might then provide a pool of propagules for microbial colonization, which is supported by the fact that microbial assemblages in ice and soil habitats are relatively similar (Kaštovská et al., 2007; Wynn‐Williams, 1990). This has recently been supported by Pessi, Lara, et al. (2018), Pessi, Pushkareva, et al. (2018), who found that cyanobacteria transported from nearby glacial environments are the main colonizers of ice‐free soil following glacier retreat. On a worldwide scale, various factors regarding long‐range dispersal of microorganisms between and across both polar regions have also been identified: Atmospheric circulation can transport spores or even cells over large distances (Elster, Delmas, Petit, & Reháková, 2007; González‐Toril et al., 2009), and marine migratory birds, which are known to cross the two hemispheres (Schlichting, Speziale, & Zink, 1978), may introduce alien strains. This supports the theory that species occurring in these habitats are opportunistic organisms with wide ecological tolerances and strong colonizing potential rather than polar specialists. In contrast, Antarctica, unlike any other region, encompasses the most isolated environment since its separation from Gondwanaland more than ten million years ago (Vincent, 2000). Therefore, if endemic species exist among microorganisms, it is very likely that they will be found in Antarctica (Chrismas, Anesio, & Sánchez‐Baracaldo, 2018; Komárek, 2015; Strunecký, Elster, & Komárek, 2011).

Identification of cyanobacteria or eukaryotic algae from BSCs of extreme environments has predominantly been based on morphological features evaluated by light microscopy and culture techniques (Campbell, Seeler, & Golubic, 1989; Flechtner, Boyer, Johansen, & DeNoble, 2002; Flechtner, Johansen, & Clark, 1998). Reference literature designed for temperate, and therefore inadequate areas (Broady & Kibblewhite, 1991; Komárek, Kopecký, & Cepák, 1999), has been the frequent resource for species identification. Members from the most abundant order Oscillatoriales, for example, which contain narrow, filamentous cyanobacteria have often been misidentified as *Phormidium* sp. (Stal & Krumbein, 1985), or were placed within the genera *Leptolyngbya* (Nadeau et al., 2001), due to cryptic morphological features. Other studies have concentrated on molecular methods (Garcia‐Pichel & Belnap, 2001; Rigonato et al., 2013; Yeager et al., 2004), including extreme polar environments (Garcia‐Pichel, López‐Cortés, & Nübel, 2001), and have revealed that former culture‐based studies missed significant proportions of the microbial diversity (Torsvik & Øvreås, 2008; Ward, Weller, & Bateson, 1990). Modern approaches illustrate repeatedly the importance of combining different methodologies: Molecular data need to be correlated with ecological and morphological data, where comparisons are necessary to update or correct the present system, especially for cyanobacteria (Komárek, 2010). For these reasons, modern analyses of microbial diversity in complex natural communities, such as BSCs, need to include polyphasic methodologies, which combine the use of traditional and molecular techniques. The efficiency of a polyphasic approach to determine the relatedness of different polar strains has been shown (Comte, Šabacká, Carré‐Mlouka, Elster, & Komárek, 2007), but there is a gap in available and correct data in databanks. For these reasons, it is complicated to continue further phylogenetic investigations on cyanobacteria in extreme cold areas.

A recent study revealed cyanobacterial diversity patterns (Rippin et al., 2018) of the Arctic and Antarctic sites included within this study but was unable to discriminate on a species level between cyanobacteria that were present in a living and active state and remnants of dead organisms. Therefore, we applied an intensive combination of denaturing gradient gel electrophoresis (DGGE), light microscopy, culturing, and sequencing of cyanobacterial isolates to compare cyanobacteria within the BSC of Arctic, Antarctic, and European Alpine sites. Insights into cyanobacterial community compositions were made to critically challenge questions regarding biogeographic aspects. As recently highlighted by the group of Chrismas et al. (2018), the applied approach contributes to genomic techniques to further our understanding of cyanobacteria in cold environments in terms of their evolution and ecology.

## 2 MATERIALS AND METHODS

### 2.1 Sampling sites

In order to cover a vast range of geographic distance with shared climatic characteristics, four BSC dominated study sites with tundra‐like biomes were selected. A brief description of the sampling sites is summarized in Table 1, and more information is given in Jung et al. (2018).

**Table 1:** Sampling sites with climate characteristics of temperature and precipitation. References for mean annual precipitation (MAP), climatic conditions, and BSC coverage are given in Jung et al. (2018)

Location	Coordinates	MAP (mm)	Elevation (m.a.s.)	*T*_min_ (°C)	*T*_max_ (°C)	BSC coverage (%)	Climate classification
Hochtor	47°04′57.50″N 12°51′01.50″E	1.800	2.500	−10	+4	>60	Alpine‐polar
Ny‐Ålesund	78°55′26.33″N 11°55′23.84″E	471	<100	−12	+5.8	90	Polar/Tundra
Geopol	78°56′58.38″N 11°28′35.64″E	471	<100	−12	+5.8	20	Polar/Tundra
Livingston	62°39′46.00″S 60°23′20.00″W	445	<100	−2.8	+4.3	20–55	Polar

### 2.2 Sampling procedure

Samples from Hochtor (Austria, Alpine) were taken during the Soil Crust International Project (SCIN) in July, 2012 (Büdel et al., 2014). Samples from Livingston Island (Antarctica) were collected during February 2015 and samples from Svalbard, Spitsbergen (Geopol and Ny‐Ålesund, Arctic) in August 2014 (Williams, Borchhardt, et al., 2017). Ten samples per site were randomly selected from areas where BSC dominated (including bryophytes, lichens, cyanobacteria, and green algae) as described in Jung et al. (2018).

### 2.3 DNA extraction

Total genomic DNA was extracted using a cetrimonium bromide (CTAB) method followed by phenol‐chloroform‐isoamyl alcohol purification adapted for BSCs (Williams, Jung, et al., 2017). This method was applied to four samples from Livingston, Geopol and Ny‐Ålesund. Six samples from Hochtor were chosen because of high levels of heterogeneity. Due to difficulties in removing contaminants from the DNA samples, a further step was included: the DNA was cleaned using the NucleSpin^®^ Gel and PCR Clean‐up Kit (Macherey‐Nagel GmbH & Co. KG) following the DNA and PCR clean up protocol. This was found to be sufficient in producing DNA of high enough quality for downstream applications. DNA was stored at −20°C until further processing.

### 2.4 Denaturing gradient gel electrophoresis (DGGE)

A nested PCR approach was utilized to amplify the DNA for denaturing gradient gel electrophoresis (DGGE). The 16S rRNA gene region was initially amplified using the primers 27F1 and 1494Rc (Neilan et al., 1997), followed by a subsequent second PCR for DGGE analysis with the primers CYA359F (with a 40‐base GC clamp) and equimolar concentrations of CYA781Ra and CYA781Rb (Nübel, Garcia‐Pichel, & Muyzer, 1997). Explicit PCR conditions are described in Williams, Jung, et al. (2017).

Denaturing gradient gel electrophoresis of the PCR products was performed on a 6% (w/v) polyacrylamide gel (40% Acrylamide/Bis solution 37.5:1, Bio‐Rad) with a 50–65% gradient formed with urea and formamide as denaturants (100% denaturing solution contained 40% v/v deionized formamide and 7 M urea), in a Ingeny Phor U‐2 system (INGENY International BV, Netherlands) containing 17 L 1× TAE buffer. Electrophoresis was run at a constant voltage of 100 V at 60°C for 16 hr, after which gels were stained with SYBR Gold^®^ (Invitrogen, USA) and visualized under a UV trans‐illuminator (UVsolo TS—Analytik Jena AG).

### 2.5 Fingerprint analysis

To analyze the community banding patterns, the fingerprinting software BioNumerics 7.6 (Applied Maths, Kortrijk, Belgium) was used to correct the images, calculate densitometric curves based on the light intensities and positions of the bands, estimate the number of bands, calculate diversity indices (Shannon‐Wiener), community evenness, and establish dendrograms as well as multidimensional scaling (MDS).

Shannon‐Wiener inde x ***H***_**SW**_ was calculated as: $H_{\mathrm{SW}}=\Sigma_{i=1}^{n}-\frac{h_{i}}{H}\log\frac{h_{i}}{H}$

Community evenness ***E*** was calculated as: $E=\frac{H_{S}W}{\ln n}$

With ***n*** as the total number of bands in the profile, ***h***_***i***_ as the light intensity of the individual band ***i,*** and ***H*** as the total intensity of all bands in the profile.

The calculation of the similarities is based on the Pearson (product‐moment) correlation coefficient (Pearson, 1926), and results in a distance matrix. The Pearson correlation is an objective coefficient which does not suffer from typical peak/shoulder mismatches, as often found when band‐matching coefficients are used.

UPGMA with arithmetic averages with the multistate categorical similarity coefficient was used to calculate the dendrograms of the DGGE gel. Using multidimensional scaling (MDS) analysis, the data of complex DGGE patterns of one sample could be reduced to one point in a three‐dimensional space. MDS does not analyze the original dataset, but the distance matrices of each DGGE using a similarity coefficient (Pearson's correlation).

### 2.6 Enrichment cultures and isolates

A maximum of 250 mg soil material was randomly picked from BSC samples and incubated in 15 ml liquid Bold's Basal Medium plus soil extract (BBM; Bischoff & Bold, 1963) overnight. The tubes were subsequently shaken and then stabilized for 30 s to allow sedimentation of debris and particles. Supernatant was decanted into a new tube and the sediment again added to 15 ml BBM. The procedure was repeated three times, all supernatant was combined resulting in a total of 45 ml BBM. This solution was centrifuged for five minutes at 1,000 rpm. The supernatant was decanted and the pellet resuspended in 250 μl of double distilled water. Light microscopy was employed to check for cyanobacterial content, and samples were subsequently transferred to solidified BBM with soil extract, BG11 and BG11_0_ media (Stanier, Kunisawa, Mandel, & Cohen‐Bazire, 1971) with ten replicates per site (four for BBM and BG11, each; two for BG11_0_). The variety of media was chosen to capture a broad diversity of cyanobacteria with different requirements. The enrichment cultures were kept in a culture cabinet at 15–17°C under a light/dark regime of 14:10 hr at a light intensity of ca. 20–50 μmol photons m^−2^ s^−1^ as described in Langhans, Storm, and Schwabe (2009) for at least 4 weeks, because these parameters guaranteed suitable growth. This can be explained by a study on vegetation mats of Svalbard that revealed an increase by about 5°C during summertime within the communities (Coulson et al., 1993), which shows that the culture conditions were comparable to the environmental conditions.

The cultures were inspected twice a week for the appearance of cyanobacteria, and colonies were transferred with a sterile metal needle to new BG11 medium agar plates. This was repeated until unialgal cultures were achieved. The growth of the colonies was frequently monitored and several subcultures were generated by further serial transfers under sterile conditions, until contamination with other cyanobacteria, green algae, or fungi was eradicated and unialgal isolates could be established.

### 2.7 PCR of isolates and sequencing

Small proportions from unialgal isolates of cyanobacterial strains were used for DNA extraction as described above with the exception of using 0.5 ml Buffer B instead of 1 ml. Extracted DNA was cleaned using the NucleSpin^®^ Gel and PCR Clean‐up Kit (Macherey‐Nagel GmbH & Co. KG), following the DNA and PCR clean up protocol. A nested PCR approach was chosen with a first PCR with the primer set of 27F1 and 1494Rc as described for DGGE and a subsequent second PCR with the primer set of CYA361f and CYA785r for cyanobacteria (Mühling, Woolven‐Allen, Murrell, & Joint, 2008), with the adaption of 61°C as annealing temperature instead of 59°C. The obtained PCR product was cleaned using the NucleSpin^®^ Gel and PCR Clean‐up Kit as before. A total of 28 samples were sequenced by Seq‐It GmbH & Co. KG (Pfaffplatz 10, 67655 Kaiserslautern, Germany), and the sequences were submitted to GenBank (Accession No. X to Y) and compared with publicly available sequences in the National Center for Biotechnology Information (NCBI) database (http://www.ncbi.nlm.nih.gov/) using the Basic Local Alignment Search Tool for Nucleotides (BLASTN) search function. A list of sequences from species publicly available on GenBank with the highest similarity to our strains is shown in supporting information (Table S1). All generated sequences will be submitted to GenBank with the project accession number PRJEB28195.

### 2.8 Phylogenetic analysis

The 16S rRNA gene sequences were aligned using the ClustalW algorithm of Mega 7 (Kumar, Stecher, & Tamura, 2016) and manually edited to remove ambiguous regions. The tree includes the most similar uncultured NCBI BLAST hit from GenBank as well as the most similar species hit for each isolated and sequenced strain. The evolutionary history was inferred by using the maximum‐likelihood method based on the Jukes‐Cantor model (Jukes, Cantor, & Munro, 1969), produced with Mega 7. The bootstrap consensus tree inferred from 500 replicates is taken to represent the evolutionary history of the taxa analyzed (Felsenstein, 1985), rooted to *Gloeobacter violacaeus* PCC 7421. A total of 398 bp were used in the final dataset. Extra sequences of *Gloeocapsa* species from GenBank were added to confirm the position of *Gloethece fuscolutea* within the phylogenetic tree. The percentage of replicate trees in which the associated taxa clustered together in the bootstrap test are shown next to the branches. Initial trees for the heuristic search were obtained automatically by applying Neighbor‐Join and BioNJ algorithms to a matrix of pairwise distances estimated using the maximum composite likelihood (MCL) approach and then selecting the topology with superior log likelihood value. All positions containing gaps and missing data were eliminated. Alternative maximum‐likelihood trees with bootstrap analyses using Paup 4.0b10 yielded similar results to trees made with Seaview 4.0 (data not shown). The same process was applied to check the phylogenetic relationship of two sequences obtained from *Oculatella* isolates.

### 2.9 Light microscopy and identification

Cyanobacterial populations were studied by light microscopy using oil immersion and a 630‐fold magnification and AxioVision software (Carl Zeiss, Jena, Germany). Appropriate taxonomic keys (Geitler, 1932; Komárek & Anagnostidis, 1998, 2005) were consulted for identification.

### 2.10 Statistical analysis

Statistics for calculated diversity index values, species richness, as well as evenness were completed using the software Statistica (Version 9.1; StatSoft Inc. 2010). The data were tested for normal distribution with a Shapiro‐Wilk test. After all data were found to be normally distributed, a one‐way ANOVA with a following Tukey post hoc test was used to look for differences between groups.

## 3 RESULTS

### 3.1 Cluster analysis dendrogram and MDS

**Figure 1:**
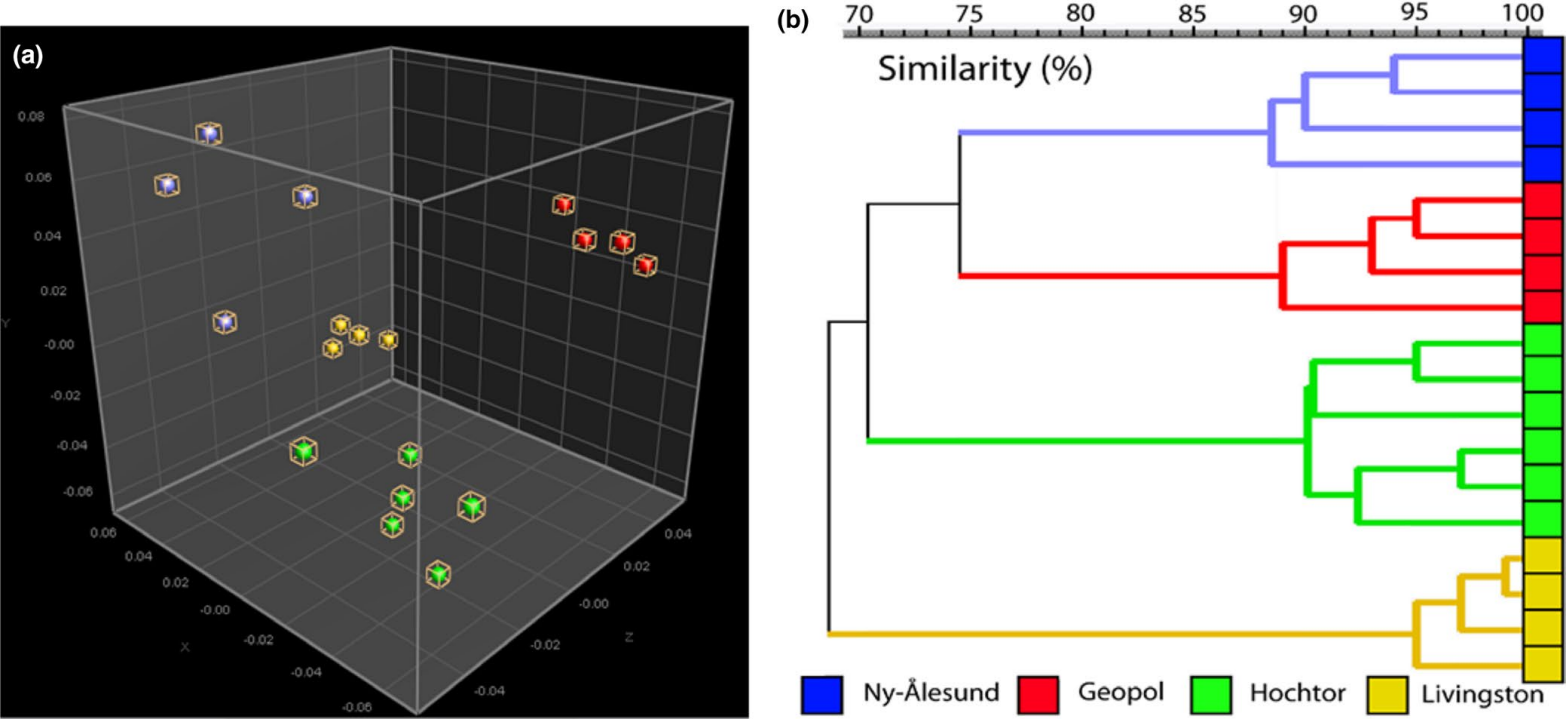
Community level fingerprint analysis. Multidimensional scaling (MDS) of DGGE banding patterns (a) and as cluster analysis (b), based on similarities according to their habitats

The MDS analysis (Figure 1a) represents cyanobacterial communities from the four different sites in the three‐dimensional plot, which grouped together according to their geographical origins, based on banding patterns and therefore their relatedness.

The calculated cluster analysis dendrogram (Figure 1b) shows four clusters based on the DGGE patterns, separating the four sampling sites based on their cyanobacterial communities. Samples of the two sites from Svalbard (Arctic) were more closely related to each other than to Hochtor (Alpine) samples. Cyanobacterial communities of Livingston (Antarctica) showed a greater divergence from the Alpine and Arctic communities.

### 3.2 Diversity

Shannon‐Wiener diversity index H_SW_, based on the DGGE banding patterns of the specific groups is an approach to estimate the diversity of microbial communities, for example, the higher H_SW_, the greater the diversity of the microbial community. A diversity index consists of two components: The total numbers of species present or species richness and the distribution of the number of individuals among those different species, called species evenness (Kennedy & Smith, 1995). The mean and the standard deviations of the Shannon‐Wiener index *H*_SW_ values, species richness, and evenness for each cyanobacterial community are listed in Table 2.

**Table 2:** Fingerprint diversity values. Mean and standard deviation of DGGE‐based calculated Shannon‐Wiener (*H*_SW_) diversity index, species richness and evenness

Sampling site	Hochtor	Geopol	Ny‐Ålesund	Livingston
Shannon‐Wiener index (*H*_SW_)	1.18 ± 0.07^a^	1.11 ± 0.07^a^	1.18 ± 0.06^a^	0.58 ± 0.06^b^
Species richness	20.33 ± 1.86^a^	11.50 ± 2.52^b^	17.25 ± 2.22^a^	8.0 ± 1.41^b^
Evenness	0.43 ± 0.02^a^	0.90 ± 0.01^b^	0.66 ± 0.01^c^	0.74 ± 0.05^d^

^a,b,c,d^Samples which are significantly different (*p* < 0.05).

The results of the calculated Shannon‐Wiener diversity index H_SW_ reveal that Hochtor and Ny‐Ålesund harbored equally the most diverse (1.18), and Livingston significantly the least diverse cyanobacterial communities (0.58 ± 0.06). Species richness values based on the number of bands in the fingerprint images support that Ny‐Ålesund (17.27 ± 2.27) and Hochtor (20.33 ± 1.86) also had the highest species richness, with Livingston showing the species poorest cyanobacterial population (8.0 ± 1.41). Community evenness values revealed that the dominance at Geopol was equally distributed among the single taxa (0.9 ± 0.01), whereas at Hochtor single taxa dominated over others (0.43 ± 0.02).

### 3.3 Species determination

**Figure 2:**
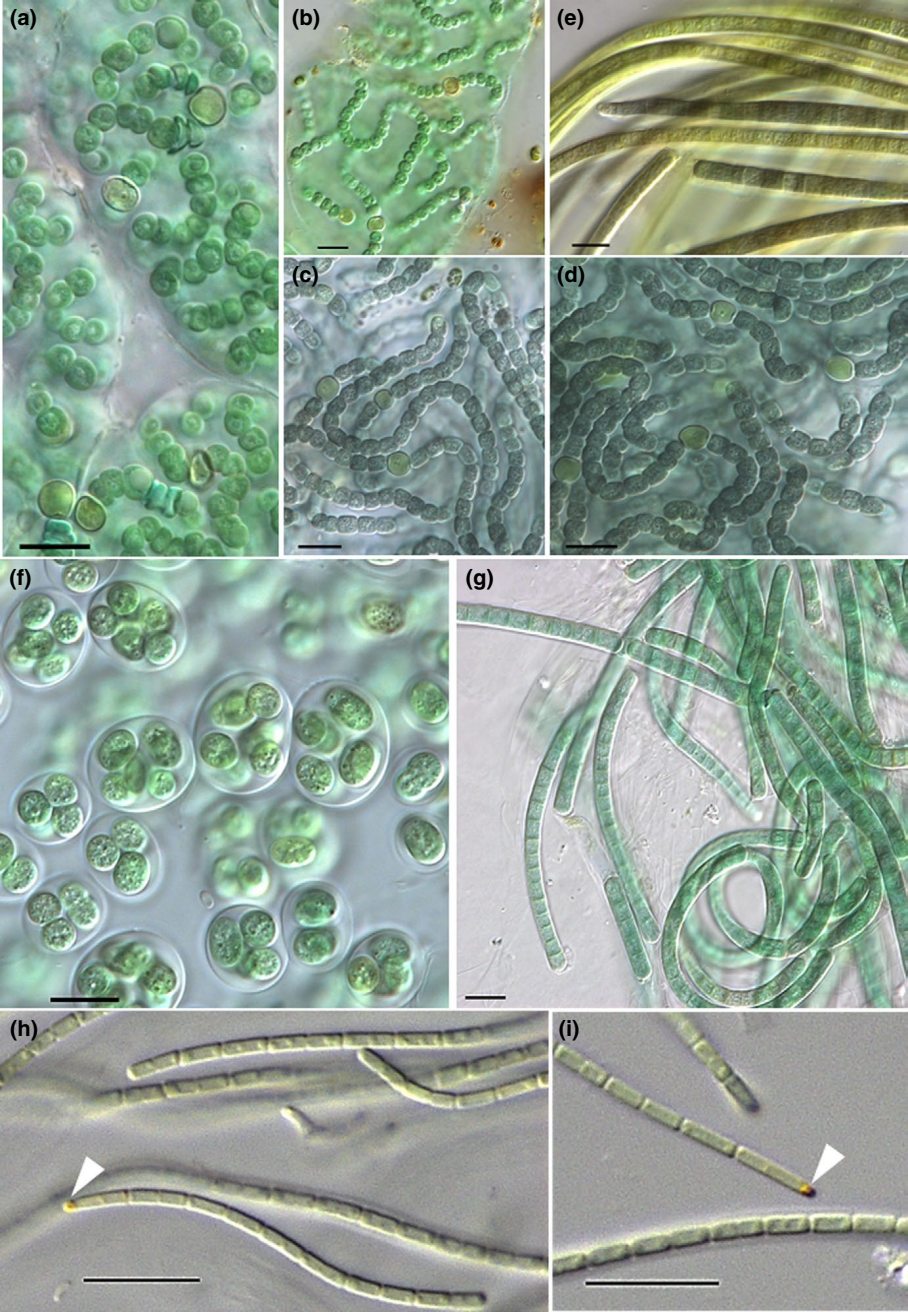
Microscopic images. (a) *Nostoc flagelliforme* from BBM culture of Hochtor, and (b) from direct microscopy of BSC from Geopol, (c) *N. edaphicum* from BG11 culture of Geopol and (d) from Ny‐Ålesund, (e) *Microcoleus vaginatus* from BG11 culture of Geopol, (f) *Gloeothece fuscolutea* from BG11_0_ culture of Hochtor, (g) *Wilmottia murrayi* from BG11 culture of Livingston, (h) and (i) *Oculatella sp*. with the reddish eyespot (white triangle) from BG11 culture of Geopol. Scale bar indicates 10 μm, 630× magnification for a‐g, 1,000× for (h) and (i)

A total of 22 taxa across all four sites could be identified by combining direct microscopy of BSC material (M) and sequencing of isolated strains (S) shown in Table 3. For some species, only the genus could be determined by light microscopy due to poor morphological features. Hochtor comprised 15 of these, Ny‐Ålesund 9, Geopol 11, and Livingston 4. Different *Nostoc* species were shared among sites (Table 3, Figure 2a–d) whereas the species *Wilmottia murrayi* (Figure 2g) and *Oculatella* sp. (Figure 2h,i) were found in single habitats only (Table 3). Based on microscopic observations of Hochtor samples, a few taxa were found to be dominant (e.g.: *Gloethece fuscolutea*,* Leptolyngbya frigida*,* Leptolyngbya antarctica,* and *Nostoc commune*), compared to others (e.g., *Stigonema, Chroococcus*), which were relatively rare. The opposite was discovered for Geopol where almost all taxa occurred in equal proportions.

**Table 3:** Species list. Identified species are listed based on light microscopy (M) as a direct observation from BSC material or culture isolates and sequencing (S)

Species	Hochtor	Ny‐Ålesund	Geopol	Livingston
*Aphanothece* sp.	M		M	
*Chroococcidiopsis* sp.	M			
*Chroococcus* sp.	M			
*Gloeothece fuscolutea*	S, M		M	
*Leptolyngbya antarctica*	S, M	S, M	S, M	
*Leptolyngbya foveolarum*	S			
*Leptolyngbya frigida*	M	S, M		
*Microcoleus vaginatus*	M	M	S, M	
*Nostoc commune*	S, M	S, M	M	M
*Nostoc edaphicum*		S, M	S, M	
*Nostoc flagelliforme*	S, M		S, M	
*Nostoc gelatinosus*				M
*Nostoc microscopicum*			S, M	M
*Nostoc pruniforme*		S, M		
*Oculatella* sp.			S	
*Oscillatoria geminata*	S			
*Phormidium autumnale*	M	M	M	
*Pseudanabaena* sp.	M	M		
*Schizothrix* sp.	M			
*Stigonema* sp.	M	M		
*Tolypothrix* sp.			M	
*Wilmottia murrayi*				M, S
Total	15	9	11	4

### 3.4 Phylogenetic tree

**Figure 3:**
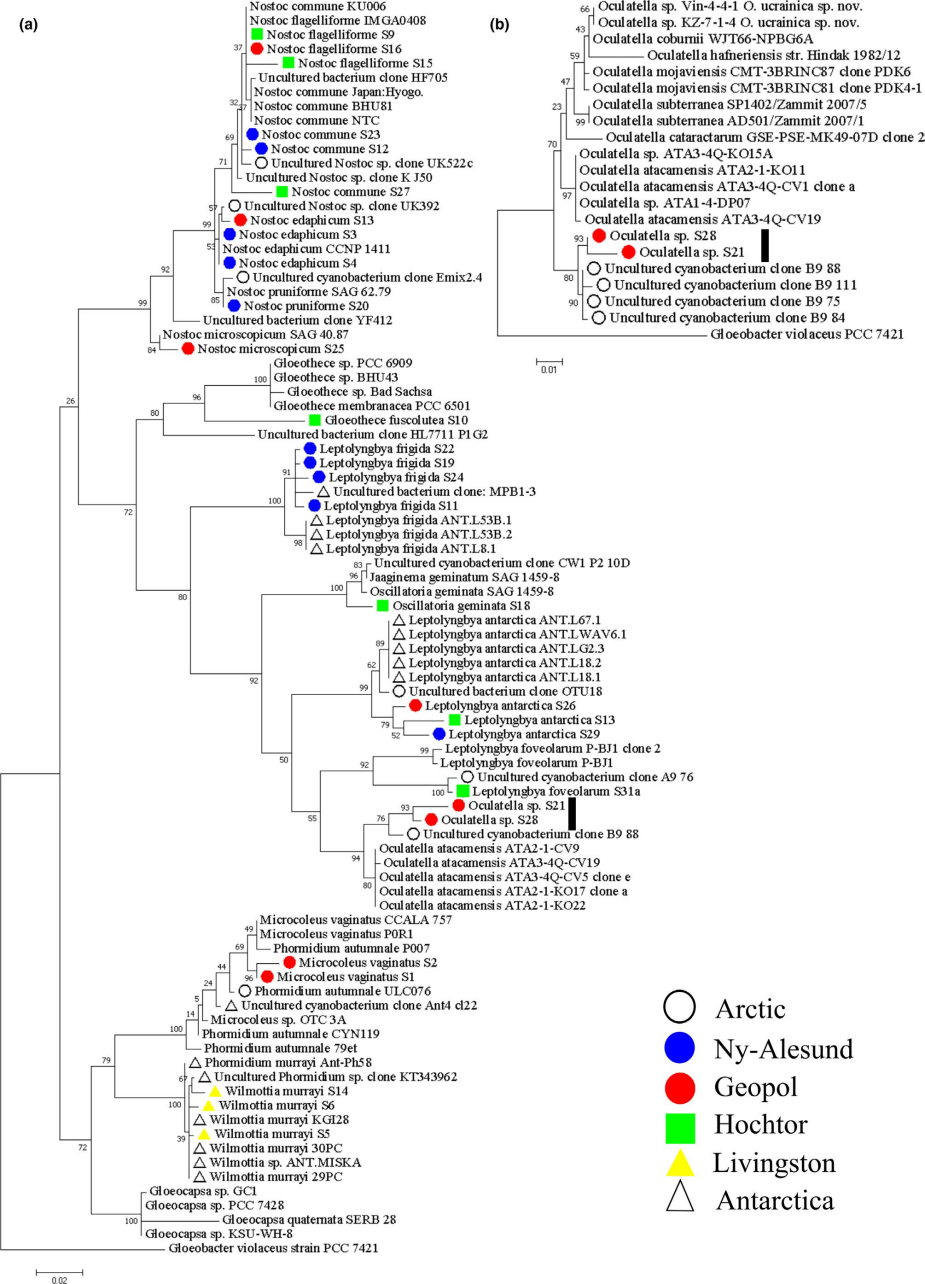
Phylogenetic maximum‐likelihood trees with bootstrap values. Shown are isolated cyanobacterial sequences and their color coded local origins together with publicly available sequences (a). The number of sequences from one species represents different isolates. The vertical black bar indicates the position of two *Oculatella* sp. strains (a, b) that were grouped in a second tree to publicly available strains of the genus *Oculatella* (b). The scale gives the number of base pare substitutions per site

An analysis of the phylogenetic sequence relationships from 28 sequences obtained from cultivated cyanobacterial isolates from living BSC proportions is represented in Figure 3a. All sequences showed high similarity with publicly available sequences (BLAST similarities above 97%), excepting *Oculatella* sp. and *Gloeothece fuscolutea* (supporting information Table S1, S2). For the latter species, sequences were unavailable in GenBank, but to clarify the position of *G. fuscolutea* sequences of *Gloecapsa*, a morphologically similar group of the order Chroococcales was added. Our strains, which were assigned morphologically to the genus *Oculatella*, joined already known species of this genus in the phylogenetic tree (Figure 3a), but formed a separate clade with uncultured sequences derived from the Arctic (Figure 3b).

## 4 DISCUSSION

To our knowledge, this is the first study in which a concentrated effort has been carried out to obtain a wide variety of cyanobacterial strains from BSCs of the Arctic (Geopol, Ny‐Ålesund), Antarctica (Livingston Island) and European Alps (Hochtor), and where a microscopic analysis combined with DGGE and 16S rRNA gene sequencing from isolated cyanobacteria has been performed. These techniques were combined as a polyphasic approach to compare cyanobacteria at community and species level in order to address biogeographic aspects as well as BSC composition in addition to previously published meta‐transcriptome data.

### 4.1 Community level: DGGE

Highly diverse ecosystems, such as BSCs, benthic mats, and soils, reveal DGGE banding patterns that are very complex to interpret. Due to several drawbacks of the method, this interpretation depends on the applied methodological workflow and has do be taken with care. Computer‐aided analyses are necessary to examine these patterns by means of fingerprint analysis. On the basis of statistical and molecular analysis (MDS, cluster analysis, DGGE), the different cyanobacterial communities were divided according to their habitats (Figure 1a,b). Differences between the cyanobacterial communities of Geopol and Ny‐Ålesund which are only 8 km apart can be explained due to the great differences seen in soil structure as demonstrated in Williams, Borchhardt, et al. (2017): While at Ny‐Ålesund normal tundra soils occur, Geopol is a rocky site with skeletal soils which limit substrate structures for microorganism colonization. Additionally, the thin soils have a reduced water holding capacity which together with cryoturbation and the action of the underlying permafrost creates major environmental factors which limits microbial growth (French & Guglielmin, 2000). On the other hand, soil properties such as pH, organic carbon, nitrogen, and phosphorous contents are very similar at Antarctica and the Arctic sites (Mann, Sletten, & Ugolini, 1986; Otero, Fernández, de Pablo Hernandez, Nizoli, & Quesada, 2013). Confocal laser scanning microscopy (CLSM) images of the BSCs from Geopol showed that these factors lead to a thin photosynthetic active layer (PAL; Jung et al., 2018). Differences between BSC functional group compositions of Geopol and Ny‐Ålesund were shown by Williams, Borchhardt, et al. (2017). However, our results show that the functional group differences (cyanobacterial crust, green algal crust, cyanolichens, chlorolichens, bryophytes) are also reflected within the single group of cyanobacteria. Williams, Borchhardt, et al. (2017) also revealed similarities between the biotic and abiotic functional groups of Geopol and Livingston Island, which also relates to the cyanobacterial species richness values (Table 2). Livingston receives higher levels of precipitation during austral summer than Svalbard in northern summer, and therefore, the vegetation is not so reliant on meltwater. This allows the hillocks, which would not be accessible to meltwater accumulation, to be abundant in fruticose lichens‐ and bryophyte‐dominated BSCs (Williams, Borchhardt, et al., 2017). In comparison, we can support previous assumptions regarding cyanobacteria as the climax vegetation stage at Hochtor (Büdel et al., 2014) and in Svalbard, where sites are not dominated by scree or polygon soils (Williams, Borchhardt, et al., 2017). Although the initial colonization and crust formation by cyanobacteria is considered as a pioneering stage of BSC development (Turicchia et al., 2005; Yoshitake, Uchida, Koizumi, Kanda, & Nakatsubo, 2010), these cyanobacteria dominated crusts can also contribute to a climax community at heavily disturbed sites (Szyja, Büdel, & Colesie, 2018; Williams, Borchhardt, et al., 2017).

The Shannon‐Wiener diversity index for the cyanobacteria was found to be almost equal for the Alpine and Arctic sites, but significantly lower (*p* < 0.05) for Livingston Island, the Antarctic location (Table 2). In contrast, the number of observed taxa, namely the number of bands between Hochtor (~20), Geopol (~12), Ny‐Ålesund (~17), and Livingston Island (~8) is different (Table 2). Both parameters combined indicate that at Hochtor single taxa occur in high abundances and therefore are dominant in the cyanobacterial community. Community evenness calculations and microscopic observations supports this result because at Hochtor the evenness is in comparison low, whereas it is high at Geopol, where all taxa seem to be equally dominant (Table 2). As access to liquid water is essential for cyanobacteria to photosynthesize (Lange, Kilian, & Ziegler, 1986), three of the sites are strongly dependent on meltwater, this may be the explanation for these results. Hochtor has the highest precipitation levels throughout the growing season, whereas in Svalbard snow melt takes place mainly at the beginning of the summer season. Communities at Ny‐Ålesund can rely on this water at least for a short period but at Geopol the coarse ground caused by the polygon formation diminishes the water holding capacity (Hodkinson, Webb, Bale, & Block, 1999), which could lead to less diverse cyanobacterial populations and arrested succession.

Hochtor harboring the most diverse cyanobacteria dominated BSC supports previous ideas. An extremely thick PAL structure was visualized by CLSM connected to high diversity for Hochtor and the opposite for Livingston Island (Jung et al., 2018). Besides water availability the light regime could also be a responsible factor, because all four sites share similar daylight times with photosynthetic active radiation (PAR) exceeding 1200 μmol m^−2^ s^−1^ (Barták, Váczi, & Hájek, 2012; Colesie, Green, Raggio, & Büdel, 2016; Xiong & Day, 2001), but with the strongest fluctuations at Hochtor (Büdel et al., 2014). The appearance of photoautotrophic organisms down to several millimeters in depth may be possible due to a diverse community composition of organisms with different adaptions regarding light regime (Belnap, Phillips, & Miller, 2004). Immobile *Nostoc* colonies for example were mainly found on top of soils where an investment in UV protection is essential. In contrast, mobile species such as *Microcoleus* spp. adapt by migrating to favorable positions within the soil (Garcia‐Pichel & Pringault, 2001). The establishment of a highly diverse cyanobacterial community composition with representatives of different light demands can lead to an increase in activity and consequently the thickness of PAL. Additionally, activity recovery of BSCs after seasonal changes may be accelerated by a vivid crust (Pushkareva, Pessi, Wilmotte, & Elster, 2015). Previous studies reporting high rates of photosynthetic activity during the snow‐free growing season for BSCs of Hochtor (Büdel et al., 2014; Raggio et al., 2017) also support this idea. The results obtained through DGGE regarding a species rich cyanobacterial community in Ny‐Ålesund and species poor communities in Antarctica are confirmed by meta‐transcriptome analysis, which revealed 67 cyanobacterial genera for the Arctic and 16 for Antarctica (Rippin et al., 2018).

### 4.2 Species Level: Light microscopy and culture derived sequences

The DGGE method applied to (cyano‐)bacteria is not without drawbacks. For example, single bands do not always represent a single organism (Sekiguchi, Tomioka, Nakahara, & Uchiyama, 2001), and bands that migrated to the same position in different lanes may consist of different bacteria (Nübel et al., 1997; Satokari, Vaughan, Akkermans, Saarela, & de Vos, 2001). The method is also based on DNA content of the soil rather than the cyanobacteria that are currently a living part of a BSC that could be detected only by RNA based techniques. Additionally, it has been shown that each DNA extraction method can result in different community profiles (Luo, Hu, Zhang, Ren, & Shen, 2007), reflected by the number and intensity of bands in the DGGE fingerprint reducing the comparability of different methods applied. Thus, the effects of universal primers, DNA extraction method, the fingerprint, and the analysis should be carefully interpreted. Nevertheless, DGGE was found to be a sufficient method because the fingerprint results were crosschecked via classical culture methods using different media. This elucidated the community composition at a species level by sequencing isolated cyanobacteria and included microscopic identification so only living components of the community were captured. Both culturing and fingerprinting methods resulted in the highest diversity at Hochtor and the lowest found at Livingston (Table 3), which corresponds with the trends found in the fingerprint diversity and species richness calculations. A similar study that applied next generation sequencing to investigate the cyanobacterial diversity stated 11 species for Hochtor with almost identical species (Williams, Loewen‐Schneider, Maier, & Büdel, 2016), supporting the validity of DGGE. The distinct species composition at each site is supported by the patterns found in the cluster analysis and MDS (Figure 1). Culturing supports the strength of DGGE as a suitable tool for rapid and highly comparative analysis of unknown natural communities (Ranjard, Poly, & Nazaret, 2000). Although there are limitations, DGGE still remains an excellent, highly reproducible, and comparatively low‐cost community analysis tool when used appropriately (Neilson, Jordan, & Maier, 2013).

Several molecular studies have focused on the cyanobacterial diversity of aquatic ecosystems in the Arctic and Antarctica (Comte et al., 2007; Strunecký et al., 2011; Taton et al., 2018), but studies focusing on the terrestrial ecosystems dominated by BSCs have been scarce (Pushkareva et al., 2015; Wood, Rueckert, Cowan, & Cary, 2008). Additionally, little is known concerning their phylogenetic affiliations, geographic distribution, physiology, and bioactive metabolites combined with the aspect of a limited number of Antarctic, Arctic or Alpine cyanobacterial strains available in culture collections. The isolation of *Wilmottia murrayi* (Figure 2g; synonym for *Phormidium murrayi*, Strunecký et al., 2011; see also sequence similarity in Figure 3a), exclusively from the Antarctic Peninsula, for example, encourages the theory of cyanobacterial endemism in Antarctica (e.g., Komárek, 2015). However, a sequence recently derived from indirect molecular data showed 100% similarity to *W. murrayi* TM2ULC130 cultured from Antarctica, but it also showed high similarities (99–100%) with sequences retrieved from China, US, Spain, Bolivia, New Zealand, and Ireland (Pessi, Lara, et al., 2018; Pessi, Pushkareva, et al., 2018). Although the vast majority of sequences belonging to this OTU currently come from Antarctica, these analyses challenge the status of its Antarctic endemicity.

Isolated Arctic *Nostoc edaphicum* and *Nostoc commune* species were highly similar to uncultured data obtained from the Arctic. The same origin between the sequences from isolates and sequences from uncultured material could be shown for *Leptolyngbya* foveolarum.

Addressing biogeographic distribution patterns, it is likely that it is easier for extreme sites that are close to habitats with moderate abiotic conditions to acquire a higher diversity due to a close and broad pool of propagules (Martiny et al., 2006). This may be applicable to for the Alpine Hochtor, which is accessible to windblown cyanobacteria or those distributed by birds. This is in contrast to Antarctica that has by far the longest history of isolation (Pointing et al., 2015; Vincent, 2000), and where cyanobacterial endemism is expected (Taton, Grubisic, Brambilla, De Wit, & Wilmotte, 2003).

Recently, the new genus *Oculatella* was described (Zammit, Billi, & Albertano 2012; Osorio‐Santos et al., 2014). Although the genus was only assigned to Mediterranean sites (Zammit et al., 2012; Osorio‐Santos et al., 2014), our site at Geopol provides the first record of this genus from the Arctic [Correction added on 24 January 2019, after first online publication: Reference citation “Zammit, Billi, & Albertano, 2012” has been added]. The photosensitive reddish “eyespot”‐like structure (oculus) at the tip of mature apical cells (Figure 2h,i), and the phylogenetic relationship places our strains within this genus, but forming an individual clade (Figure 3b). The sequences of our strains show highest similarities to uncultured strains isolated from Svalbard, Arctic, thus representing a clade so far restricted and different from the other ‘desert’ members of this genus. This suggests the discovery of a new *Oculatella* species, which maybe endemic. Additionally, two new *Oculatella* species were recently found outside arid ecosystems which were described from BSCs of the coastline and chalk outcrops of Ukraine (Vinogradova, Mikhailyuk, Glaser, Holzinger, & Karsten, 2017). These findings highlight inventory incompleteness and lack of knowledge regarding the ecological niches of many terrestrial cyanobacteria. This raises questions regarding the eco‐physiological potential of many cold adapted cyanobacteria like *Wilmottia murrayi* and the apparently broader‐than‐thought ecological tolerance of the genus *Oculatella*. Correct assignments of 16S rRNA sequences to publicly available sequences of species with unambiguous morphological characteristics such as the “eyespot” of *Oculatella* species proves the validity of the approach applied in this study. Although multigene analysis such as a combination of 16S and ITS is preferred, we could demonstrate that sequencing parts of the 16S in combination with light microscopy contributes to recent investigation pipelines for cyanobacteria of cold biomes (Chrismas et al., 2018).

A closer look at cold environment assigned cyanobacteria reveals that to date only a few 16S rRNA gene sequences are available from mostly uncultured Antarctic or Arctic cyanobacteria (e.g. Casamatta, Johansen, Vis, & Broadwater, 2005; Jungblut et al., 2005; Nadeau et al., 2001). Nevertheless, these studies have shown that many sequences from Antarctic or Arctic cyanobacteria form distinct clusters that are at least assigned to cold biomes. A clone‐library analyses indicate that three taxa previously identified as Antarctic endemics (*Phormidum priestleyi*,* Leptolyngbya frigid,* and *L. antarctica*) were more than 99% similar to sequences from the Canadian High Arctic (Jungblut, Lovejoy, & Vincent, 2010), which is also the case for the two *Leptolyngbya* species identified in this study. In 2010, the group of Strunecký was unable to show 16S rDNA‐based genetic clusters according to the north‐ or south pole origin of *Phormidium*‐like strains, which also seems to be the case here. Beside the arguments for cyanobacterial endemism and cold environment assigned cyanobacteria derived from aquatic strains, we can confirm these aspects of the debate with terrestrial cyanobacteria derived from BSCs. Finally, our results provide a link between genotypic and phenotypic features by revealing the efficiency of a polyphasic approach, which allows a better understanding of cyanobacteria diversity, biogeographic distribution patterns, and corrects current database entries.

## 5 OUTLOOK

Upcoming studies will contain in situ photosynthetic long term monitoring to assess eco‐physiological parameters of cyanobacteria dominated BSCs from the same ecosystems. Additional laboratory experiments with isolated species and transcriptomics will reveal their ecological importance, biotechnological potential, and gene expression under extreme environmental conditions.

## CONFLICT OF INTEREST

The authors declare that they have no conflict of interest.

## AUTHOR CONTRIBUTION

PJ processed all molecular analysis and prepared the manuscript, LBW took the samples and helped to prepare the manuscript, isolation was carried out by MS and BB guided all work.

## ETHICAL STATEMENT

This article does not contain any studies with human or animals performed by any of the authors.

## Data Availability

The authors declare that all data generated or analyzed during this study are included in this article. Sequences can be found in GenBank under the project accession number PRJEB28195.
