# Supplementary material for: Strong in combination: Polyphasic approach enhances arguments for cold‐assigned cyanobacterial endemism
Source: Microbiologyopen. 2018 Sep 21;8(5):e00729. doi: 10.1002/mbo3.729 (PMC6528576; doi:10.1002/mbo3.729)
Supplement: Supplementary file 1 [file MBO3-8-e00729-s001.docx]

**STab. 1:** Sequence similarity. Generated sequence number are given with their closest NCBI genbank species hit in percent similarity. Congruence between the origin of the isolated species and its corresponding most similar publicly available NCBI blast hit is indicated in grey.

| **Sequence** | **Organism** | **Highest species hit** | **accession no.** |
| --- | --- | --- | --- |
| S2* | *Microcoleus vaginatus* | 97% *Phormidium autumnale* ULC076 | MH118740* |
| S1* | *Microcoleus vaginatus* | 97% *Phormidium autumnale* ULC076 | MH118740* |
| S9 | *Nostoc flagelliforme* | 98% *Nostoc flagelliforme* IMGA0408 | EU178143 |
| S16* | *Nostoc flagelliforme* | 97% *Nostoc flagelliforme* IMGA0408 | EU178143 |
| S15 | *Nostoc flagelliforme* | 97% *Nostoc flagelliforme* IMGA0408 | EU178143 |
| S27 | *Nostoc commune* | 98% *Nostoc commune* UTEX 584 | AY218833 |
| S12* | *Nostoc commune* | 98% *Nostoc commune* ACSSI 035 | KY283071 |
| S23* | *Nostoc commune* | 98% *Nostoc commune* ACSSI 035 | KY283071 |
| S20* | *Nostoc pruniforme* | 98% *Nostoc pruniforme* SAG 62.79 | KM019941 |
| S3* | *Nostoc edaphicum* | 97% *Nostoc edaphicum* CCNP 1411 | KJ161445 |
| S4* | *Nostoc edaphicum* | 97% *Nostoc edaphicum* CCNP 1411 | KJ161445 |
| S13* | *Nostoc edaphicum* | 97% *Nostoc edaphicum* CCNP 1411 | KJ161445 |
| S25* | *Nostoc microscopicum* | 98% *Nostoc microscopicum* SAG 40.87 | GQ287653 |
| S14** | *Wilmottia murrayi* | 98% *Wilmottia murrayi* 30PC | KY288995** |
| S6** | *Wilmottia murrayi* | 98% *Wilmottia murrayi* 30PC | KY288995** |
| S5** | *Wilmottia murrayi* | 98% *Wilmottia murrayi* 30PC | KY288995** |
| S10 | *Gloeothece fuscolutea* | 92% *Gloeothece* sp. BHU43 | KY129703 |
| S19* | *Leptolyngbya frigida* | 97% *Leptolyngbya frigida* ANT.L8.1 | AY493610** |
| S22* | *Leptolyngbya frigida* | 97% *Leptolyngbya frigida* ANT.L8.1 | AY493610** |
| S11* | *Leptolyngbya frigida* | 97% *Leptolyngbya frigida* ANT.L8.1 | AY493610** |
| S24* | *Leptolyngbya frigida* | 97% *Leptolyngbya frigida* ANT.L8.1 | AY493610** |
| S26* | *Leptolyngbya antarctica* | 98% *Leptolyngbya antarctica* ANT.L18.1 | AY493607** |
| S29* | *Leptolyngbya antarctica* | 98% *Leptolyngbya antarctica* ANT.L18.1 | AY493607** |
| S13 | *Leptolyngbya antarctica* | 98% *Leptolyngbya antarctica* ANT.L18.1 | AY493607** |
| S31a* | *Leptolyngbya foveolarum* | 97% *Leptolyngbya* sp. Uher 2000/2452 | HM018689 |
| S28* | *Oculatella sp.* | 95% *Oculatella atacamensis* ATA2-1-CV9 | KF761584 |
| S21* | *Oculatella sp.* | 94% *Oculatella atacamensis* ATA2-1-CV9 | KF761584 |
| S18 | *Oscillatoria geminata* | 98% *Jaaginema geminatum* SAG 1459-8 | KM019979 |

* Arctic origin

** Antarctic origin
