# Supplementary material for: Strong in combination: Polyphasic approach enhances arguments for cold‐assigned cyanobacterial endemism
Source: Microbiologyopen. 2018 Sep 21;8(5):e00729. doi: 10.1002/mbo3.729 (PMC6528576; doi:10.1002/mbo3.729)
Supplement: Supplementary file 2 [file MBO3-8-e00729-s002.docx]

**STab. 2:** Sequence similarity. Generated sequence number are given with their closest NCBI genbank uncultured hit in percent similarity and their origin. Congruence between the origin of the isolated species and its corresponding most similar publicly available uncultured NCBI blast hit is indicated in grey.

| **Sequence** | **Organism** | **Highest uncultured hit** | **accession no.** |
| --- | --- | --- | --- |
| S2* | *Microcoleus vaginatus* | 96% Uncult. bacterium UOXA-f10 | EU869664** |
| S1* | *Microcoleus vaginatus* | 97% Uncult. cyanobacterium Ant4 cl22 | KM052830** |
| S9 | *Nostoc flagelliforme* | 97% Uncult. bacterium HF705 | KF037277 |
| S16* | *Nostoc flagelliforme* | 97% Uncult. bacterium HF705 | KF037277 |
| S15 | *Nstoc flagelliforme* | 97% Uncult. bacterium HF705 | KF037277 |
| S27 | *Nostoc commune* | 96% Uncult. *Nostoc* sp. KJ50 | JQ007762 |
| S12* | *Nostoc commune* | 97% Uncult. Nostoc sp. UK522c | JQ007801* |
| S23* | *Nostoc commune* | 97% Uncult. Nostoc sp. UK522c | JQ007801* |
| S20* | *Nostoc pruniforme* | 97% Uncult. cyanobacterium Emix2.4 | JX887895* |
| S3* | *Nostoc edaphicum* | 97% Uncult. Nostoc sp. UK392 | JQ007798* |
| S4* | *Nostoc edaphicum* | 97% Uncult. Nostoc sp. UK392 | JQ007798* |
| S13* | *Nostoc edaphicum* | 97% Uncult. Nostoc sp. UK392 | JQ007798* |
| S25* | *Nostoc microscopicum* | 95% Uncult. bacterium YF412 | KF037820 |
| S14** | *Wilmottia murrayi* | 97% Uncult. *Phormidium* sp. KT343962 | KT343962** |
| S6** | *Wilmottia murrayi* | 98% Uncult. *Phormidium* sp. KT343962 | KT343962** |
| S5** | *Wilmottia murrayi* | 97% Uncult. *Phormidium* sp. KT343962 | KT343962** |
| S10 | *Gloeothece fuscolutea* | 89% Uncult. bacterium HL7711 P1G2 | KC896654 |
| S19* | *Leptolyngbya frigida* | 97% Uncult. bacterium MPB1-3 | AB630385** |
| S22* | *Leptolyngbya frigida* | 97% Uncult. bacterium MPB1-3 | AB630385** |
| S11* | *Leptolyngbya frigida* | 97% Uncult. bacterium MPB1-3 | AB630385** |
| S24* | *Leptolyngbya frigida* | 97% Uncult. bacterium MPB1-3 | AB630385** |
| S26* | *Leptolyngbya antarctica* | 97% Uncult. bacterium OTU18 | LC103292* |
| S29* | *Leptolyngbya antarctica* | 97% Uncult. bacterium OTU18 | LC103292* |
| S13 | *Leptolyngbya antarctica* | 96% Uncult. bacterium 9d7 | KM263186 |
| S31a* | *Leptolyngbya foveolarum* | 97% Uncult. cyanobacterium A976 | AM940672* |
| S28* | *Oculatella sp.* | 96% Uncult. cyanobacterium B9 88 | AM940918* |
| S21* | *Oculatella sp.* | 96% Uncult. cyanobacterium B9 88 | AM940918* |
| S18 | *Oscillatoria geminata* | 95% Uncult. cyanobacterium CW1 P2 10D | KC110354 |

* Arctic origin

** Antarctic origin
